# The role of context in identifying linkages between SDG 2 (food) and SDG 6 (water)

**DOI:** 10.1007/s11625-022-01158-3

**Published:** 2022-05-30

**Authors:** Han Su, Maarten S. Krol, Rick J. Hogeboom

**Affiliations:** 1 Multidisciplinary Water Management Group, Faculty of Engineering Technology, University of Twente, Horst Complex Z223, P.O Box 217, 7500 AE Enschede, The Netherlands; 2 Water Footprint Network, Drienerlolaan 5, 7522 NB Enschede, The Netherlands

**Keywords:** Sustainable Development Goals (SDGs), SDG linkages, SDG targets, Voluntary National Reviews (VNRs), Text analysis, Food–water nexus

## Abstract

The Sustainable Development Goals (SDGs) of the United Nation’s Agenda 2030 are formulated to promote the development of integrated, multisectoral policies that explicitly consider linkages across SDGs. Although multiple recent studies have tried to identify linkages across SDGs, the role of contextual factors in identifying SDG linkages is neither well described nor understood. For the case of SDG 2 and SDG 6, this study aims to (i) identify linkages—at country and SDG target level—through the application of various quantitative and qualitative identification methods, and (ii) explore contextual factors to explain the differences across identified linkages. Hereto, we first conducted a text analysis of 195 Voluntary National Reviews (VNRs) reported by 159 countries from 2016 to 2020. Next, we synthesized linkages reported by previous qualitative studies and conducted a quantitative (correlation) analysis on the UN’s SDG database. Last, we compared identified linkages across methods, paying special attention to the role of context. From the text analysis, we identified 221 country-specific linkages between 25 SDG target pairs and observed that countries increasingly report SDG linkages in their VNRs over time. Comparing text analysis, existing qualitative studies, and our quantitative correlation analysis, we found substantial differences between the number and nature of linkages identified. These differences can be explained in part by methodological considerations, but to a significant extent also by contextual factors, such as project design, technology application, phase of interventions, and project scale. We conclude by discussing the strengths and limitations of the methods involved, and suggestions for future studies.

**Supplementary Information:**

The online version contains supplementary material available at 10.1007/s11625-022-01158-3.

## Introduction

One of the biggest challenges that humanity is currently facing is how to boost prosperity for all within the means of the planet. To address this challenge, the United Nations General Assembly formulated an ambitious development agenda comprised of 17 Sustainable Development Goals (SDGs) (UNGA 2015). Covering social, economic, and environmental dimensions of sustainable development, these global goals are to be achieved by 2030. Since the SDGs are formulated in relatively abstract terms, they have been operationalized through 169 targets and 231 unique indicators (also called IAEG-SDGs) (UNSD 2020a). From its inception, the SDGs are intended to be viewed as a collective rather than as a set of siloed efforts for development (ESCAP 2015). The SDGs thus call for the development of integrated, multisectoral policies that explicitly take into consideration linkages across all SDGs, such as synergies and trade-offs between the various SDG targets.

Several methods have been proposed to identify SDG linkages, ranging from descriptive or qualitative, to quantitative methods (Breuer et al. 2019; Mainali et al. 2018; Miola et al. 2019). Qualitative methods include linguistic approaches (cf. Lim et al. 2018); literature review (cf. Flörke et al. 2019; Fuso Nerini et al. 2017; McCollum et al. 2018; Velis et al. 2017); systems thinking (cf. ESCAP 2017); expert judgment (cf. Griggs et al. 2017; Nilsson et al. 2018; Singh et al. 2018); and content or text analysis (cf. Le Blanc et al. 2017; Vladimirova and Le Blanc 2016). Quantitative methods, on the other hand, include correlation analysis (cf. Fonseca et al. 2020; Kroll et al. 2019; Pradhan et al. 2017; Wang et al. 2020; Zhou and Moinuddin 2017); principal component analysis (cf. Cling et al. 2020); Bayesian network analysis (cf. Requejo-Castro et al. 2020); system dynamic modeling (cf. Collste et al. 2017; Pedercini et al. 2019); and computable general equilibrium modeling (cf. Banerjee et al. 2019). While these different methods inevitably lead to different linkage results, additional differences arise from the various levels at which these studies have defined SDG linkages, such as goal–goal (Fonseca et al. 2020), goal–target (Flörke et al. 2019), or target–target level (Zhou and Moinuddin 2017). What these studies do have in common is that they typically identify synergies more frequently than trade-offs (ESCAP 2016; Fader et al. 2018; Pradhan et al. 2017).

Linkages are also context-dependent. In this study, we take context to mean the characteristics of countries or projects undertaken to achieve one or more SDG targets, including particular project designs, phase of intervention, scale of projects, status or availability of natural resources, climate, geography, technology application, demographics, and other socio-economic factors (Griggs et al. 2017; Mainali et al. 2018; McCollum et al. 2018). As an example, regarding the contextual factor technology application, one can think of conventional farming versus organic farming. Conventional agriculture promotes the expansion of agricultural productivity (SDG 2.3) and will likely generate a trade-off with improved water quality (SDG 6.3) or increased water availability (SDG 6.4). A similar expansion of agriculture in organic agriculture, on the other hand, may create synergies with said SDG targets (ESCAP 2016).

Despite the general support for the importance of contextual factors, their role in the identification of linkages has not been well understood—especially beyond case studies or for global scale assessments (Breuer et al. 2019; Nilsson et al. 2018). Comparing identified linkages across various methods helps in the understanding of their roles (Mainali et al. 2018). This study aims to identify SDG linkages for countries—at the target level—using various qualitative and quantitative methods and explain those differences found using contextual factors. We take SDG 2 (zero hunger) and SDG 6 (clean water and sanitation) as SDGs of focus for our analysis. First, we identified linkages based on text analysis of the Voluntary National Reviews (VNRs), in which countries report on their progress toward achieving the SDGs. Second, we characterized other linkages by reviewing qualitative identification methods applied in previous studies and conducting a widely used quantitative (correlation) analysis on the UN’s SDG database. Last, we compared linkages identified across these text analysis, qualitative, and quantitative methods, paying special attention to the role of context to explain the comparison.

## Materials and methods

**Fig. 1:**
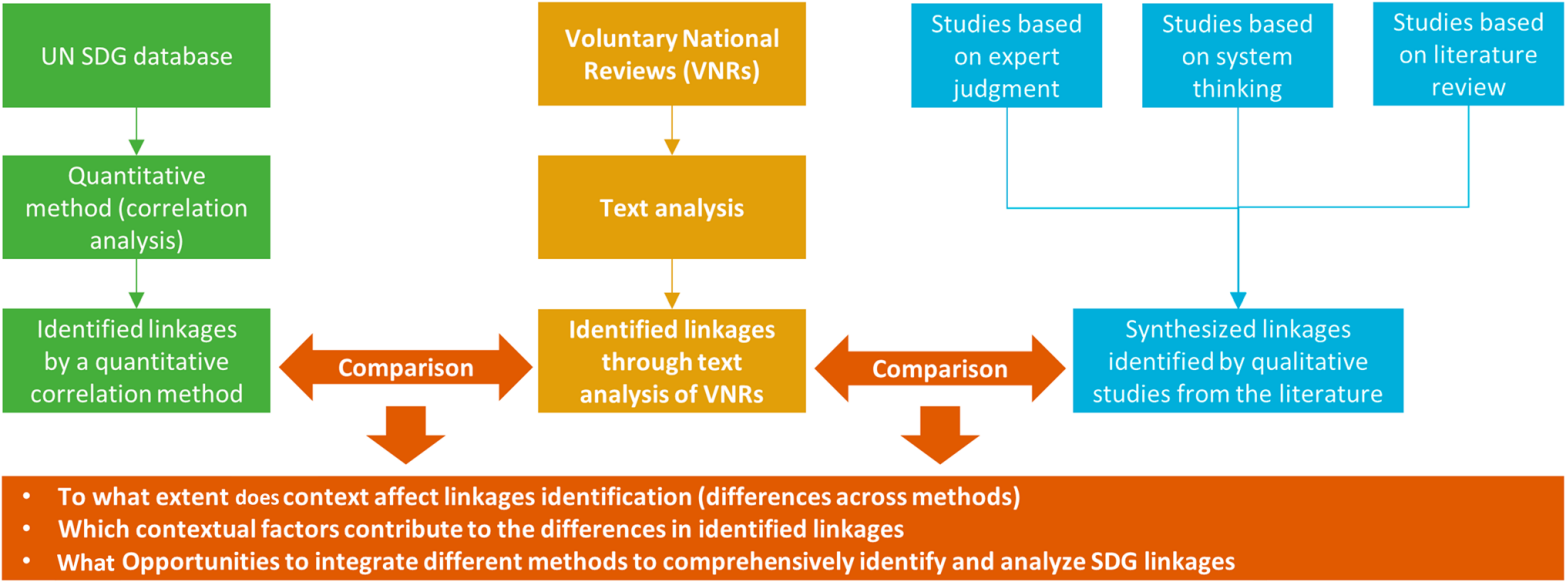
Overview of the research design

In this study, we identified SDG linkages at the target and country level using three different methods. First, we identified linkages through text analysis of VNRs. Next, we synthesized linkages identified by existing qualitative studies from the literature. Last, we identified linkages by applying a quantitative correlation method. Linkages distilled from the VNRs are country-specific and are assumed to adequately capture contextual factors influencing SDGs. Linkages identified were then compared between text analysis and synthesized linkages, and between text analysis and correlation analysis. We tried to explain the differences in identified linkages to address the role of context by discussing contextual factors using both the VNRs and literature (Fig. 1).

In this paper, we use linkage as both country-specific and non-country-specific, depending on whether the country information is available or not. Country-specific linkage means that one interaction between a target pair identified in two countries will be counted as two country-specific linkages.

### SDG linkages based on text analysis of the VNRs

We applied text analysis to Voluntary National Reviews (VNRs) to identify SDG linkages within their country-specific context. The VNR is a voluntary report that reviews progress on national implementation of the SDGs and which is prepared for the annual High-level Political Forum on Sustainable Development (HLPF) meeting (DESA 2018). In their VNRs, countries illustrate how the SDGs are incorporated into national policies, track the achievement of and progress toward each goal and target, and list major projects made or planned for each goal and target (DESA 2018, 2019). Typically, a VNR also describes challenges, synergies and trade-offs encountered, and lessons learned. VNRs report SDGs at various levels of granularity, from project or local level to national level, and from immediate project consequence to long-term policy effect. Between 2016 and 2020, 164 countries released a total of 200 VNRs, with some countries releasing two or three reports. Our premise is that the VNRs provide a comprehensive overview based on which we can identify SDG linkages in a country-specific context.

We subjected all 200 VNRs to text analysis. Among the 200 VNRs, 56 VNRs are non-English (28 French, 24 Spanish, three Arabic, one Russian) and thus needed translation. We used Google Translate for the translation of these VNRs to English. Google Translate achieves an average performance score of 5.2 on a 0–6 scale, versus an average of 5.4 that humans score for similar tasks (Wu et al. 2016), implying it is an adequate tool for the job. Because of several typesetting issues encountered for five VNRs, we ended up with a data set comprised of 195 VNRs, from 159 countries [see [S1] for a description of excluding issues].

Since we focused on the linkages between food (SDG 2) and water (SDG 6), each VNR was searched with particular attention for (i) sections on SDG 2 and SDG 6; (ii) sections with the terms “synthesis”, “highlight”, “introduction”, “national background”, “national context”, “lessons learned”, “national challenges”, “summary” or related terms in its heading; and (iii) paragraphs that contain the term “water”. Since we wanted to find the text about both food and water, we searched for “water” instead of “food” because “water” has fewer synonyms and is easier to locate.

After an initial reading, potentially indicative sections were highlighted from which SDG linkages could be identified. To minimize subjectivity and bias in the identification process as much as possible and to maintain consistency across the VNRs, the following criteria were applied to determine if a linkage was identified. [S2] provides several examples to illustrate the above text analysis procedure.

1. The indicative sections could be located in a text element (e.g., a sentence, paragraph, or figure) or a logical sequence of text elements (e.g., multiple text paragraphs all covering the same policy or project).
2. Either the SDG target itself or its associated keywords or both were explicitly mentioned within the text element. Keywords for each target (Table 1) were derived from definitions of respective indicators provided by IAEG-SDGs (2020) (Tier Classification for Global SDG Indicators, 17 July 2020).
3. The type of linkage (i.e., a synergy or a trade-off) could be inferred from the text element itself.

**Table 1:** SDG targets of SDG 2 and SDG 6 (IAEG-SDGs 2020)

Goal 2. End hunger, achieve food security and improved nutrition and promote sustainable agriculture
2.1 By 2030, end hunger and ensure access by all people, in particular the poor and people in vulnerable situations, including infants, to safe, nutritious and sufficient food all year round
2.2 By 2030, end all forms of malnutrition, including achieving, by 2025, the internationally agreed targets on stunting and wasting in children under 5 years of age, and address the nutritional needs of adolescent girls, pregnant and lactating women and older persons
2.3 By 2030, double the agricultural productivity and incomes of small-scale food producers, in particular women, indigenous peoples, family farmers, pastoralists and fishers, including through secure and equal access to land, other productive resources and inputs, knowledge, financial services, markets and opportunities for value addition and non-farm employment
2.4 By 2030, ensure sustainable food production systems and implement resilient agricultural practices that increase productivity and production, that help maintain ecosystems, that strengthen capacity for adaptation to climate change, extreme weather, drought, flooding and other disasters and that progressively improve land and soil quality
2.5 By 2020, maintain the genetic diversity of seeds, cultivated plants and farmed and domesticated animals and their related wild species, including through soundly managed and diversified seed and plant banks at the national, regional and international levels, and promote access to and fair and equitable sharing of benefits arising from the utilization of genetic resources and associated traditional knowledge, as internationally agreed
2.a Increase investment, including through enhanced international cooperation, in rural infrastructure, agricultural research and extension services, technology development and plant and livestock gene banks to enhance agricultural productive capacity in developing countries, in particular least developed countries
2.b Correct and prevent trade restrictions and distortions in world agricultural markets, including through the parallel elimination of all forms of agricultural export subsidies and all export measures with equivalent effect, in accordance with the mandate of the Doha Development Round
2.c Adopt measures to ensure the proper functioning of food commodity markets and their derivatives and facilitate timely access to market information, including on food reserves, to help limit extreme food price volatility
Goal 6. Ensure availability and sustainable management of water and sanitation for all
6.1 By 2030, achieve universal and equitable access to safe and affordable drinking water for all
6.2 By 2030, achieve access to adequate and equitable sanitation and hygiene for all and end open defecation, paying special attention to the needs of women and girls and those in vulnerable situations
6.3 By 2030, improve water quality by reducing pollution, eliminating dumping and minimizing release of hazardous chemicals and materials, halving the proportion of untreated wastewater and substantially increasing recycling and safe reuse globally
6.4 By 2030, substantially increase water-use efficiency across all sectors and ensure sustainable withdrawals and supply of freshwater to address water scarcity and substantially reduce the number of people suffering from water scarcity
6.5 By 2030, implement integrated water resources management at all levels, including through transboundary cooperation as appropriate
6.6 By 2020, protect and restore water-related ecosystems, including mountains, forests, wetlands, rivers, aquifers and lakes
6.a By 2030, expand international cooperation and capacity-building support to developing countries in water- and sanitation-related activities and programmes, including water harvesting, desalination, water efficiency, wastewater treatment, recycling and reuse technologies
6.b Support and strengthen the participation of local communities in improving water and sanitation management

One linkage of a pair of targets may be mentioned twice or more by one country about multiple aspects, and these aspects were recorded during text analysis. For linkages counting, each linkage in one country was counted a maximum of once. Considering the identifying uncertainties, the direction of linkages were recorded and counted based on policy intention but not included during the comparison.

### SDG linkages from qualitative methods

We synthesized SDG linkages from four existing studies that all used qualitative methods to identify linkages: ESCAP (2016), Griggs et al. (2017), Parikh et al. (2020), and UNWater (2016). These studies were selected based on their comprehensive scope and inclusion of causal descriptions for each linkage. Moreover, each study covers linkages between food (SDG 2) and water (SDG 6) at the target level. We assumed these qualitative studies mainly strived for context-free generic linkages. Comparing the identified linkages from text analysis and these qualitative studies aided our understanding of how contextual factors enable or disable the possibility of linkages. In terms of the type of qualitative methods applied, Griggs et al. (2017) and UNWater (2016) use expert judgment, while ESCAP (2016) employs systems thinking and Parikh et al. (2020) a literature review. ESCAP (2016) and Griggs et al. (2017) distinguish both type and driver and response in each linkage and Griggs et al. (2017) additionally measure the strength of each linkage.

### SDG linkages from quantitative methods

We employed a correlation analysis to the identified linkages through quantitative means besides the two qualitative methods described above. Quantitatively derived linkage databases are available, e.g., from Pradhan et al. (2017), Zhou and Moinuddin (2017), and Zhou et al. (2021). Two concerns prevented us from using the datasets directly: a limited number of countries for Zhou et al. (2021) and raw data availability for Pradhan et al. (2017). Raw data are necessary to track differences between linkages in our subsequent analysis step (see “Comparing linkages identified and exploring contextual factors”), to show trends in sub-indicators of targets, and to investigate which contextual factors may contribute to the trends.

For our quantitative analysis, we performed a correlation analysis on the UN’s SDG database (UNSD 2020b) (retrieved on 17 May 2020). This database covers 60 sub-indicators for all 16 targets of SDG 2 and SDG 6 across 257 countries or regions around the world from 1995 to 2019, except target SDG 2.4 (sustainable agriculture) because of massive data needed to assess the sustainability of agriculture. Similar to other SDG databases, not all these sub-indicators were available for every country. Data coverage varies among sub-indicators, with relatively larger coverage for SDGs 2.1, 2.2, 6.1, and 6.2. Following Pradhan et al. (2017) and Zhou and Moinuddin (2017), we applied correlations at the sub-indicator level, e.g., at one sub-indicator from SDG 2 and one from SDG 6, where linkages may exist according to qualitative studies, and considered all sub-indicator combinations. We required at least four data points to calculate a Spearman correlation coefficient. We dropped all the sub-indicators that remained unchanged.

We labeled the linkage as a synergy or a trade-off if the Spearman correlation coefficient was larger than 0.6 or smaller than − 0.6 in conjunction with a *p* value below 0.05 (Pradhan et al. 2017); in other cases where the correlation was not strong or significant enough, we labeled the linkage as non-classified. Whether a strong and significant linkage constitutes a synergy or trade-off was inferred from our interpretation of the meaning of the sub-indicator pairs, rather than from the sign of the correlation coefficient, as the coefficient sign does not necessarily suggest an improvement when the sub-indicator value increases (e.g., SDG 2.1.1 ‘number of undernourished people’).

### Comparing linkages identified and exploring contextual factors

After the three different methods were applied, we compared the identified linkages across the methods. During the comparison, we excluded the direction of linkages because the direction was inferred from the mechanism of the linkages and when we looked into the specific description of the linkages, the direction became less important. Thus, we relied on the comparison of the linkages’ description instead of direction. During the comparison, the linkages were considered to be nondirectional.

We compared linkages identified by the text analysis to those synthesized from existing qualitative studies. To find the relevant contextual factors, we compared how each linkage was described in the qualitative studies and the VNRs. Both qualitative studies and VNRs provided explanations for linkages, e.g., using certain technology or implementing certain policies on an SDG target. We compared these explanations for some examples and identified contextual factors to explain the differences.

Next, we compared linkages identified by the text analysis and by the quantitative method. We relied on VNRs and literature searching to identify contextual factors. We only included those target pairs where correlation analysis at the sub-indicator level identified a linkage and where the VNR text analysis identified a target level linkage for the same country. We checked how these linkages and sub-indicators were described in VNRs. This helped us to confirm whether the target level linkages and sub-indicator level linkages from the two methods were writing about the same aspect. If the VNRs could not explain the sub-indicator changes, we then additionally searched the literature, using the country name and keywords associated with the respective sub-indicators. The search ended once the literature could explain the differences between the VNRs description and sub-indicator value changes in the UN’s SDG database. For example, for one country, there may be a correlation analysis between SDG 2.1.1 and SDG 6.2.1, indicating a different linkage compared to the linkage between SDG 2.1 and SDG 6.2 from the text analysis. We would then go through the country’s VNRs again to make sure, in the text analysis, that the target level linkages were about the sub-indicator SDG 2.1.1 and SDG 6.2.1. If so, we checked how the VNRs described SDG 2.1.1 and SDG 6.2.1 and which contextual factors affected their changes. If the VNRs could explain the sub-indicator changes and the VNRs indicated a different linkage, then the VNRs could explain why the correlation analysis and the text analysis were different. Otherwise, we searched for additional literature until we could explain the changes of SDG 2.1.1 and SDG 6.2.1 using the contextual factors. We then knew why the VNRs indicated one linkage while the values of SDG 2.1.1 and SDG 6.2.1 indicated another.

## Results

### Linkages identified by text analysis

We identified 221 country-specific linkages across 25 SDG target pairs using text analysis on 195 VNRs from 85 countries, from both developing and developed countries (Table 2) [detailed results can be found in S3]. Table 3 provides several examples of key linkages identified between SDG 2 and SDG 6 at a target level. Since the target level linkages may involve multiple aspects, the same target pair could appear more than once in Table 3, such as SDG 2.4 and SDG 6.3. For each target pair, the specific aspects mentioned in VNRs can be found in [S3]. Note, in Tables 2 and 3, the target pairs are nondirectional, but in [S3], the 221 country-specific linkages are directional.

**Table 2:** Linkages identified by text analysis and how these linkages are identified by the synthesis of qualitative studies and correlation analysis

Linkages identified by text analysis between	How the linkages was identified by:	
	The synthesis of qualitative studies	Correlation analysis
SDG 2.1 and SDG 6.1 (S)	S	S and T
SDG 2.1 and SDG 6.2 (S)	S	S
SDG 2.1 and SDG 6.4 (S)	T	–
SDG 2.2 and SDG 6.1 (S)	S	S, T, and N
SDG 2.2 and SDG 6.2 (S)	S	S, T, and N
SDG 2.2 and SDG 6.a (S)	–	–
SDG 2.3 and SDG 6.1 (S and T)	T	–
SDG 2.3 and SDG 6.2 (S)	S and T	–
SDG 2.3 and SDG 6.3 (S and T)	T	–
SDG 2.3 and SDG 6.4 (S and T)	T	–
SDG 2.3 and SDG 6.5 (S)	S	–
SDG 2.3 and SDG 6.6 (S and T)	S and T	–
SDG 2.3 and SDG 6.b (S)	–	–
SDG 2.4 and SDG 6.1 (S)	–	–
SDG 2.4 and SDG 6.2 (S)	S	–
SDG 2.4 and SDG 6.3 (S)	S	–
SDG 2.4 and SDG 6.4 (S)	S	–
SDG 2.4 and SDG 6.5 (S)	S	–
SDG 2.4 and SDG 6.6 (S)	S	–
SDG 2.4 and SDG 6.a (S)	S	–
SDG 2.4 and SDG 6.b (S)	S	–
SDG 2.5 and SDG 6.6 (S)	S	–
SDG 2.a and SDG 6.3 (S)	–	–
SDG 2.a and SDG 6.4 (S)	–	–
SDG 2.a and SDG 6.5 (S)	–	–

The linkages are nondirectional. S: synergies,T: trade-offs, N: non-classified

**Table 3:** Example of key linkages and associated driven contextual factors from text analysis where the example mechanism is reported by at least two countries

	Example mechanism	Example county	Resulting linkages between
Project design	Support vulnerable or rehabilitated communities with both clean drinking water and sanitation and food and agricultural production materials	Bhutan	SDG 2.1 and SDG 6.1 (S)
			SDG 2.1 and SDG 6.2 (S)
			SDG 2.3 and SDG 6.2 (S)
	Establish a farm cooperative where (small-scale) farmers are offered knowledge about environmentally friendly practices, facilities, and market access to save water or improve water quality at the same time	Bangladesh Ecuador	SDG 2.3 and SDG 6.4 (S)
			SDG 2.3 and SDG 6.3 (S)
			SDG 2.4 and SDG 6.3 (S)
			SDG 2.4 and SDG 6.4 (S)
Technology application	Harvest rainwater for drinking water and to support agricultural production	Saudi Arabia	SDG 2.3 and SDG 6.1 (S)
	Recycle water for use in agricultural production (as an additional water resource and to increase climate adaptability)	Malta	SDG 2.3 and SDG 6.3 (S)
			SDG 2.4 and SDG 6.3 (S)
	Use alternate wetting and drying irrigation to save water while simultaneously increasing agricultural productivity or avoiding yield reduction	Vanuatu	SDG 2.3 and SDG 6.4 (S and T)
			SDG 2.4 and SDG 6.4 (S)
	Use a weather-based farm management system to both increase climate adaptability and inform water use efficiency potential	Panama	SDG 2.4 and SDG 6.4 (S)
	Promote hydroponic farming to save water, increase economic returns, offer job opportunities, and increase agricultural sustainability	Qatar	SDG 2.3 and SDG 6.4 (S)
			SDG 2.4 and SDG 6.4 (S)
			SDG 2.a and SDG 6.4 (S)
	Use solar pumps and gravity irrigation to improve agricultural production conditions and save water	Seychelles	SDG 2.4 and SDG 6.4 (S)
			SDG 2.3 and SDG 6.4 (S)
	Promote organic farming to support simultaneously sustainable water use and sustainable agriculture	Bulgaria	SDG 2.4 and SDG 6.4 (S)
Causality	Agriculture expansion leading to higher water stress	Armenia	SDG 2.3 and SDG 6.4 (T)
	Agricultural expansion and the intensive use of fertilizer and pesticides leading to water pollution and ecosystem degradation; Efficient use of fertilizer and pesticides leading to water pollution reduction	Uruguay	SDG 2.3 and SDG 6.3 (T)
			SDG 2.4 and SDG 6.3 (S)
			SDG 2.4 and SDG 6.6 (S)
	Increasing access to clean drinking water, sanitation and hygiene contribute to malnutrition reduction via reducing contamination in tap water and infectious diseases	Kyrgyzstan	SDG 2.2 and SDG 6.1 (S)
			SDG 2.2 and SDG 6.2 (S)
	Adjust crop structure to water-saving crops to save water and increase climate adaptability	Sri Lanka	SDG 2.4 and SDG 6.4 (S)
			SDG 2.4 and SDG 6.6 (S)
	Protect water ecosystem and reduce water pollution to ensure sustainable fisheries	Algeria	SDG 2.4 and SDG 6.3 (S)
			SDG 2.4 and SDG 6.6 (S)
	Research helps to find solutions to concurrently increase water use efficiency and agricultural productivity	United Arab Emirates	SDG 2.3 and SDG 6.4 (S)
			SDG 2.4 and SDG 6.4 (S)

The linkages are nondirectional. S: synergies T: trade-offs

Most linkages concern a synergy rather than a trade-off, as out of the 221 country-specific linkages, 209 (94.6%) indicate a synergy and 12 (5.4%) a trade-off. The text analysis of VNRs confirms the previous findings that more synergies can be found than trade-offs (Miola et al. 2019; Pradhan et al. 2017). The fact that more synergies were identified by text analysis may be explained by two reasons. First, VNRs may be biased towards reporting synergies because they focus primarily on describing planned projects that assume a positivist future perspective, rather than evaluating previously implemented projects that may bring to light failures or trade-offs as well. This bias is illustrated by the fact that out of the 136 country-specific linkages that describe planned projects, 124 are indeed phrased in synergistic terms. Second, countries may intrinsically prefer to report on synergies rather than on trade-offs, as even out of the 85 linkages that are related to implemented projects, 75 concern a synergy versus ten a trade-off.

**Fig. 2:**
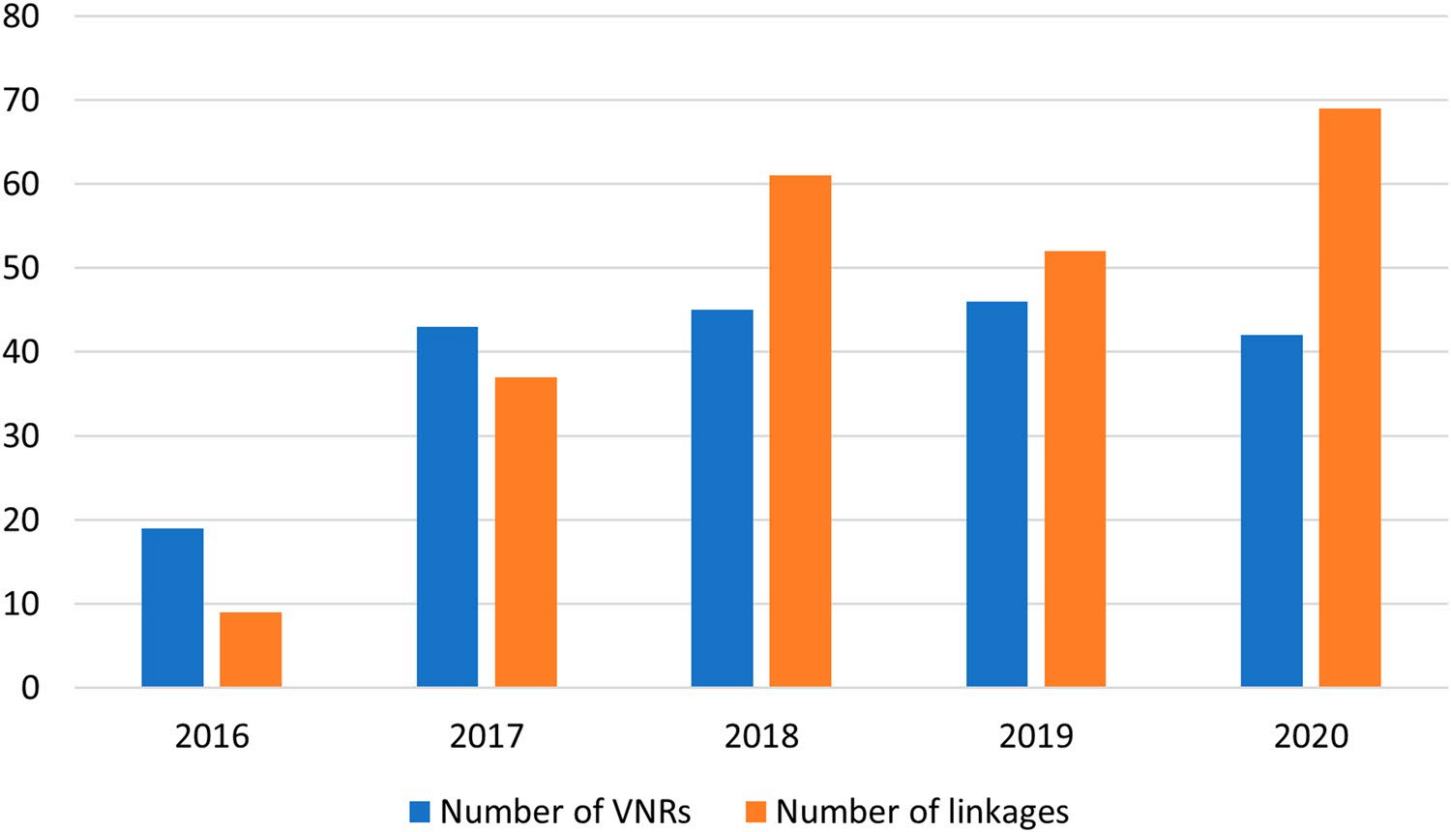
Number of VNRs and identified linkages from text analysis from 2016 to 2020

We also observed an upward trend from 2016 to 2020 in the number of linkages identified from VNRs (Fig. 2). In 2020, VNRs reported 1.67 linkages between targets of SDGs 2 and 6 per country on average, which is up by a factor of three from the 0.47 linkages identified in 2016. This increase may be explained by the writing guidelines of the VNRs that increasingly encourage countries to report on SDG linkages (DESA 2018, 2019, 2020). The trend shows a minor dip from 2018 to 2019, which may be a side effect of the specific reporting theme that was selected in 2019. Where food was highlighted in 2017 (Eradicating poverty and promoting prosperity in a changing world, SDGs 1, 2, 3, 5, 9, 11, 17) and water in 2018 (Transformation towards sustainable and resilient societies, SDGs 6, 7, 11, 12, 15, 17), 2019’s theme (Empowering people and ensuring inclusiveness and equality, SDGs 4, 8, 10, 13, 16, 17) was less strongly correlated to SDGs 2 or 6.

### Synthesized linkages from qualitative studies and linkages identified by correlation analysis

We synthesized 24 linkages from the four qualitative studies (Table 4) including sixteen synergies and eight trade-offs. Linkages from individual qualitative studies are diverse. None of the linkages were reported by all four studies at the same time.

**Table 4:** A synthesis of linkages between food (SDG 2) and water (SDG 6) at target level from qualitative studies

SDG 2	SDG 6	Source of study			
		Griggs et al. (2017)	ESCAP (2016)	UNWater (2016)	Parikh et al. (2020)
2.1	6.1	Synergy		Synergy	
2.1	6.2	Synergy		Synergy	Synergy
2.1	6.3			Trade-off	
2.1	6.4			Trade-off	
2.1	6.6			Trade-off	
2.2	6.1	Synergy	Synergy	Synergy	
2.2	6.2	Synergy		Synergy	Synergy
2.2	6.b		Synergy		
2.3	6.1	Trade-off			
2.3	6.2	Trade-off		Synergy	Trade-off and Synergy
2.3	6.3	Trade-off		Trade-off	
2.3	6.4	Trade-off	Trade-off	Trade-off	
2.3	6.5			Synergy	
2.3	6.6	Trade-off	Synergy	Trade-off	
2.4	6.2			Synergy	Synergy
2.4	6.3	Synergy	Synergy	Synergy	
2.4	6.4		Synergy	Synergy	
2.4	6.5		Synergy	Synergy	
2.4	6.6	Synergy	Synergy	Synergy	
2.4	6.a		Synergy		
2.4	6.b		Synergy		
2.5	6.6		Synergy		

The linkages are nondirectional

Through our correlation analysis, 11 sub-indicators from SDG 2 and eight sub-indicators from SDG 6 met the minimal data requirement covering 162 countries. Countries may only have part of these sub-indicators available. These sub-indicators mainly came from SDGs 2.1, 2.2, 2.5, 6.1, 6.2, and 6.6. With the available data, we identified 3147 linkages at the sub-indicator level; 726 linkages are first classified negative based on the sign of the correlation coefficient, 780 positive, and 1641 non-classified. These sub-indicator level linkages indicate the target level linkage between SDG targets 2.1 and 6.1, 2.1 and 6.2, 2.2 and 6.1, 2.2 and 6.2, and 2.5 and 6.6. This is not a particularly large number given the size and scope of the UN database used. One explanation for this relatively low number is that we required a minimum of four data points per sub-indicator pair for a potential linkage to be eligible. Since data coverage varied substantially among sub-indicators, several sub-indicator pairs did not meet this criterion. Furthermore, many targets in SDG 2 and SDG 6 are about either basic food or water needs. Since these targets are typically already achieved in developed countries, their sub-indicators remain unchanged over time. We excluded unchanged sub-indicators for which it is not possible to calculate the Spearman correlation coefficient, leaving fewer data available for correlation analysis.

Detailed results of the correlation analysis can be found in [S4].

### Comparing linkages identified by text analysis and qualitative methods

We observed considerable differences in the synergies identified by text analysis and qualitative methods. Only 18 (72%) out of 25 synergistic target pairs identified in the VNR text analysis are also described in previous studies that used qualitative identification methods (Table 2). Descriptions of the remaining seven synergistic target pairs could not be retrieved from the selected sources. More synergies from the text analysis were not only due to our limited inclusion of qualitative studies, but also the VNRs mentioned more project design and technology applications with country-specific characteristics. For example, the use of recycled water to generate linkages between SDG 6.3 and SDG 2.3 was not reported by synthesized linkages. Whereas, the VNR of the State of Palestine described a synergy through increasing the use of recycled water (SDG 6.3) to improve the access to water as a productive resource for irrigation (SDG 2.3) (GSP 2018); and the VNR of Malta reported a synergy for farmers increasing their income (SDG 2.3) by re-engineering plants to be suitable for irrigation with treated wastewater (SDG 6.3) (GRMt 2018). Similarly, organizing farmers via a cooperative club was not reported by synthesized linkages, but could generate a synergy between SDG 2.3 (technology support) and SDG 6.4 (sustainable water use) as indicated by the VNRs of Argentina (GAR 2017), Bangladesh (GPRB 2020), and Switzerland (GSC 2016). We also noticed that the project scale was different concerning the project design and technology application. The VNRs often report on specific projects for specific target groups, whereas qualitative studies report on macro-level long-term effects.

We found all four trade-offs through the text analysis that were reported in previous qualitative studies as well (Table 2). Moreover, both methods explicitly describe contextual factors under which the trade-off occurs, including which policies are governing, which technologies are applied, or the local environmental conditions. Qualitative studies reported four more trade-offs (between SDGs 2.1 and 6.3, 2.1 and 6.4, 2.1 and 6.6, 2.3 and 6.2) that were not identified by the text analysis of the VNRs (Table 3). This difference may be due to existing VNRs focusing more on planned projects that tend to underreport trade-offs.

### Comparing linkages identified by text analysis and quantitative methods

Overlapping linkages from text analysis and correlation analysis results only in four target pairs, 14 target level country-specific linkages, and 208 sub-indicator level country-specific linkages, across 11 countries for comparison (Table 5). A country was included in our comparison when it had linkages from the correlation analysis and text analysis. This limited the number of linkages available for inclusion. We noticed that many countries have sufficient sub-indicator level data available, but if the VNR text analysis did not include the same linkage or vice versa, it was still excluded from comparison. A large quantity of unmatched linkages indicates the overestimation of the correlation analysis, and conversely, the underestimation of the text analysis (see “Methodological differences” for more information).

**Table 5:** Comparison of the number of country-specific linkages identified between text analysis and quantitative analysis

		Croatia	Guinea	Indonesia	Kyrgyzstan	Malawi	Mauritania	Mozambique	Nepal	Peru	Timor-Leste	Uganda
2.1	6.1				*S = 6* *N = 0* *T = 0*				***S = 4*** ***N = 0*** ***T = 2***			***S = 2*** ***N = 0*** ***T = 4***
2.1	6.2	**S = 6** **N = 0** **T = 0**			*S = 8* *N = 0* *T = 0*							
2.2	6.1								***S = 6*** ***N = 9*** ***T = 3***	**S = 18** **N = 0** **T = 0**		
2.2	6.2		***S = 0*** ***N = 12*** ***T = 0***	***S = 0*** ***N = 12*** ***T = 6***		***S = 9*** ***N = 18*** ***T = 9***	***S = 0*** ***N = 18*** ***T = 0***	***S = 4*** ***N = 16*** ***T = 4***	***S = 9*** ***N = 9*** ***T = 0***		***S = 0*** ***N = 12*** ***T = 0***	

S, N and T are the number of sub-indicator level synergies, non-classified linkages, and trade-offs identified from correlation analysis, respectively. Bold marking indicates that text analysis and correlation analysis yield the same linkage; italics unrelated aspects; and bolditalics dissimilar linkages. In this table, we have four target pairs (four rows), 14 target level country-specific linkages (three in the first row, two in the second and third rows, respectively, and seven in the last row), and 208 sub-indicator level linkages (for example six sub-indicator level linkages in the first row for Nepal). The detailed dataset is available in [S5]

From the 14 target level linkages identified by both text analysis of the VNRs and the correlation analysis, only two refer to similar linkages. Two other linkages seemed to match, but upon further scrutiny, they related to different aspects of the given targets. The remaining ten overlapping linkages were different altogether. Detailed comparison results can be found in [S5], where we describe the project scale and the phase of the intervention of the linkages (at target level), changes in the sub-indicator values, and the potential factors that could explain observed differences based on the VNRs and the literature survey.

Differences in identified linkages can be classified into three broad categories, and for each one, contextual factors can help interpret the differences found. The first category of differences in overlapping linkages arises from a lack of consideration of social-economic conditions by the correlation analysis which are considered in the text analysis. For example, in Malawi, a trade-off between access to basic handwashing facilities (SDG 6.2 sub-indicator) and stunted child growth (SDG 2.2 sub-indicator) was suggested by correlation analysis, as both sub-indicators reduced in conjunction. In contrast, both text analysis and pairwise correlation analysis on other associated sub-indicators suggested a synergy between SDG 6.2 and SDG 2.2. According to the VNR of Malawi (GRMw 2020), the reduction in handwashing services was due to a lack of water and maintenance and did not lead to an observed increase in stunted children. Stunting is rather caused by open defecation and infectious diseases, food insecurity, and unhealthy diet (UNICEF 2018).

The second category of differences in identified linkages occurs where SDG sub-indicators associated with a given SDG target were correlated in an uninformative pair combination. Although a pairwise analysis of sub-indicators is a necessary step in the correlation analysis to avoid correlating completely random sub-indicator combinations, some sub-indicator pairs are simply uninformative. This limitation of the correlation analysis was previously observed by Zhou and Moinuddin (2017). Consequently, some differences between overlapping linkages can be attributed to such uninformative sub-indicator combinations that have no connection to reality. For example, in Nepal, correlation analysis found that undernourishment (SDG 2.1) and malnutrition (SDG 2.2) each yielded a trade-off with the improvement of drinking water services in an urban area (SDG 6.1). Although undernourishment and malnutrition could be linked with drinking water services in some (indirect) ways, the VNRs provided valuable contextual information on this linkage in Nepal, by describing how undernourishment (SDG 2.1) and malnutrition (SDG 2.2) mainly occur in rural areas, while drinking water services improvement (SDG 6.1) is mainly of concern in an urban setting (GN 2020). Combining ‘rural’ with ‘urban’ sub-indicators is thus not informative for linkage identification.

The third category comprises differences in temporal focus in the phase of the intervention and the project scale across methods. The VNRs, to a large extent, discuss planned or recently initiated projects, while correlation analysis was based on historic and evaluation data on already implemented policies and projects. Backcasting and forecasting seem able to explain differences found for the case of Indonesia, for example, where text analysis indicates a synergy between SDG 2.2 and SDG 6.2, while correlation analysis at the sub-indicator level yielded a mix of both synergies and trade-offs. Similarly, in Malawi, we observed differences in linkages identified by text analysis at the local project level, where correlation analysis yielded the same linkage at the national level.

We noticed that both text analysis and correlation analysis occasionally identified a lose-lose situation as a synergy, which is counter to how synergy is typically interpreted, although we did not observe a difference between overlapping linkages in this regard. This is for example the case for water use efficiency (SDG 6.4) and productivity of rural communities (SDG 2.3) in South Africa, where the VNRs described that the two targets could work in a synergetic fashion, but that this realization is a challenge to address (GRSA 2019). Similarly, for the linkage between drinking water service (SDG 6.1) and food security (SDG 2.1) in Uganda, correlation analysis identified a synergy because both associated sub-indicator values were worsening. Again, this lose–lose situation can be explained by socio-economic conditions, because the reduction in both food security (SDG 2.1) and drinking water service (SDG 6.1) largely results from large numbers of refugees living near Uganda’s capital of Kampala as pointed out by the VNR of Slovenia (GRS 2020). Distinguishing the lose–lose situation from win–win synergies in futures studies, like in Zhou and Moinuddin (2021), would help to avoid unexpected consequences in achieving SDGs.

## Discussion

### Uncertainties and limitations in linkages identified

We identified linkages using text analysis and correlation analysis and retrieved linkages previously identified by studies that applied qualitative methods. Regarding the text analysis of VNRs, we searched text elements on SDG 2 and SDG 6 which contained the keyword “water”. This may have caused us to overlook related content on, e.g., aquatic ecosystems, irrigation, drought, or other food and agriculture-related elements that do not explicitly use the term water. However, we aimed to capture as much relevant information from the VNRs as possible, by also including the summary and introduction sections of the VNRs. We continued searching the VNR manually if it pointed to potentially useful information on SDGs 2 or 6. Even though we aimed to be transparent and clearly outline the procedures and criteria applied during our text analysis, some degree of subjectivity will remain in our identification of linkages through this method.

Regarding the retrieval of linkages from literature, we reviewed milestone global studies, but it was not feasible to include all (regional) literature on linkages identified by qualitative methods. Since studies with global linkage coverage typically apply general rules for linkage identification and may overlook specific country or local characteristics, qualitative methods focusing on local issues may have the ability to include more contextual factors. Meta-analysis of local qualitative studies based on a global database could also provide more contextual factors.

Regarding the correlation analysis, it would be possible to include multiple databases to identify more linkages. The databases on SDGs grow rapidly. More data enable more identified linkages and thus more contextual factors could be revealed when compared to text analysis.

### Methodological differences

Differences in identified linkages could often be explained by referring to contextual factors, however inherent methodological differences underlie these observations. For example, the VNRs may only reflect the particular concerns but may not cover all the linkages existing in an individual country. Qualitative methods at the global scale tend to describe more generic linkages (context-free) that are reproducible across countries instead of case specific linkages. They focus more on causalities and describe projects or technologies that are aimed at macro-level change. In contrast, correlation analysis relies on monitored sub-indicators that refer to historic policies or interventions that occurred in the real world rather than causality. It draws on the time series and focuses on the country level. Theoretically, the dynamics of each sub-indicator correlation analysis included all relevant contextual factors. However, in reality, it is difficult to unpack all contextual information from mere sub-indicator values. When no sound mechanism could be found behind a correlation, a “spurious correlation”, or an overestimation of linkages, must be considered.

**Fig. 3:**
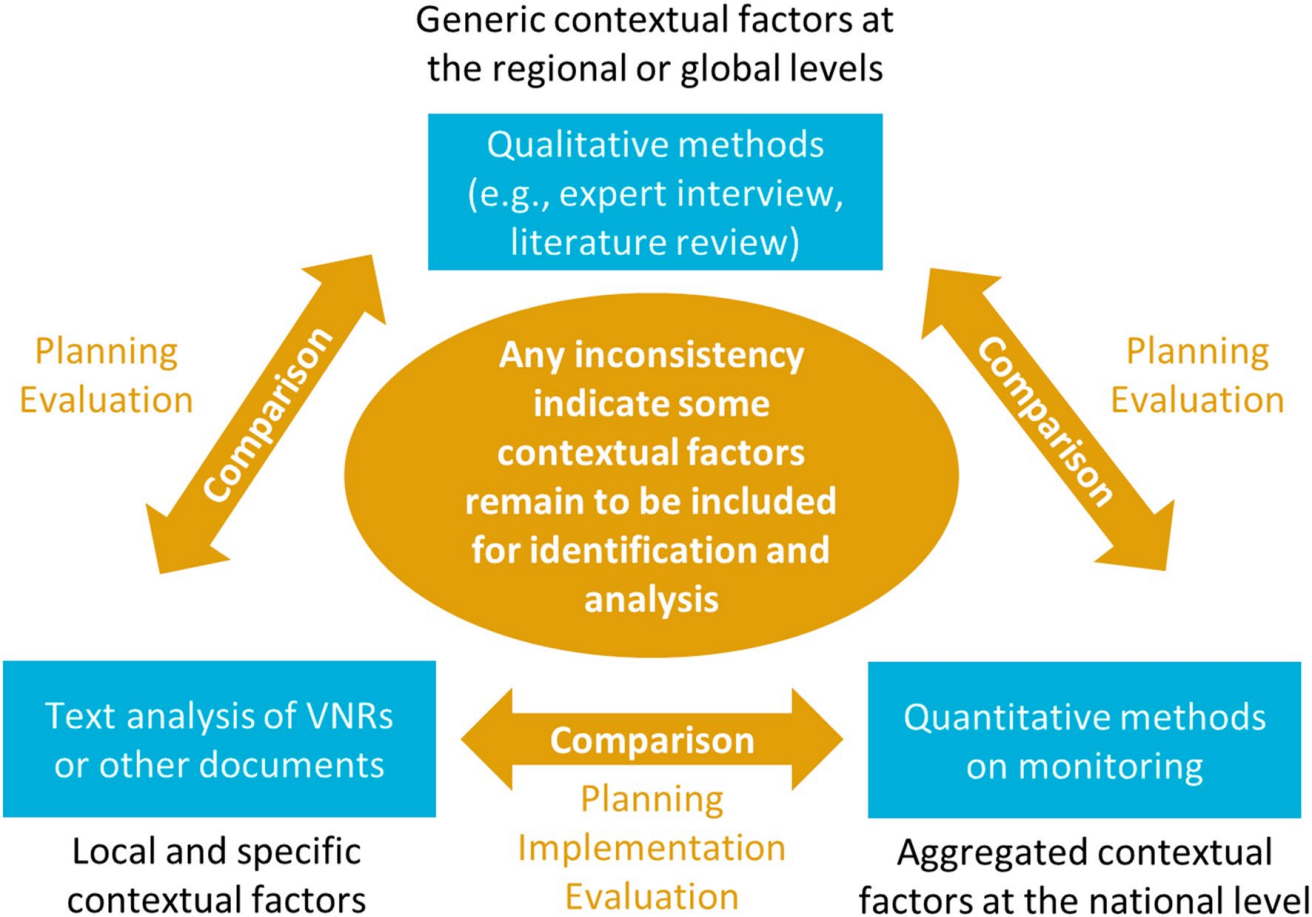
Relations between different identification methods at various stages of policy-making

These inherent methodological differences imply that there is no single method that can include all relevant contextual factors across all scales and SDG levels. Figure 3 aims to guide practitioners in their choice of method by relating the various identification methods to various stages of policy-making. During the planning stages, qualitative methods could provide a preliminary scan to identify both assumed existing and aimed linkages. Assumed existing linkages could be further validated using both text analysis of VNRs, or other official documents and correlation analysis. If differences are encountered, this may indicate that certain contextual factors have not been adequately considered. To achieve planned linkages, text analysis of other countries’ VNRs could be beneficial to inform policy and project design, e.g., how contextual factors lead to synergy in some countries while a trade-off in other countries for the same target pair. Other quantitative methods such as system dynamic modeling may further assist in conducting scenario analysis for planned SDG linkages. During the evaluation phase, and to a lesser extent the implementation phase, correlation analysis seems an appropriate method to test whether planned linkages are being realized or not. Differences could be further validated by text analysis. Again, observed inconsistencies may point to contextual factors affecting the achievement of planned linkages. Throughout the processes, any lesson learned can be used to update the knowledge database comprising qualitative studies.

### The way forward

Based on our research, we suggest that future studies attempting to identify SDG linkages might focus on the sub-indicator level to capture as many relevant contextual factors as possible. Since SDG goals or targets cover many relevant aspects and sub-indicators, they aggregate over underlying sub-indicator dynamics. SDG 6.6, for example, includes both surface water ecosystems and groundwater, but project designs, technologies, or causalities that benefit or relate to surface water do not necessarily apply to groundwater. Moreover, keyword identification during text analysis or sub-indicator selection during correlation analysis may become more feasible at the sub-indicator level because of its narrower scope.

We also propose future research needs to understand the causality and direction of linkages in a more comprehensive way. We found that for several target pairs, e.g., sustainable water use (SDG 6.4) and sustainable agricultural production (SDG 2.4) or providing food (SDG 2.1) and drinking water (SDG 6.1) for vulnerable groups, it is hard to tell which is the driver and which is the response target by the current definition of driver and response [cf. ESCAP 2016; Griggs et al. 2017). Moreover, direction of a linkage may reverse depending on the policy intention. For example, Poland’s non-point agricultural pollution controls (SDG 6.3) are expected to drive the implementation of sustainable agriculture (SDG 2.4) (GRP 2018), whereas in Finland the implementation of sustainable agriculture (SDG 2.4) should result in pollution reduction (SDG 6.3) (GRF 2020). In addition to better understanding the role of contextual factors, increasing our knowledge of linkage causality and its implications has the potential to improve SDG policy-making.

## Conclusion

Reaching the UN’s SDGs requires careful consideration of connections between interlinked SDGs. Since the role of context in the identification of SDG linkages is poorly understood in current literature, we set out to explore that role for the case of SDG 2 on food and SDG 6 on water. We compared different qualitative and quantitative methods for the identification of linkages and found major differences between country-specific linkages at the target level, which could be explained by a method’s inclusion or exclusion of particular contextual factors, such as project designs, technology applications, and project scale. Qualitative methods, including text analysis, typically have a strong capacity to include contextual factors, but since they generally take a forward-looking perspective, they may overly emphasize desired synergetic outcomes. Quantitative methods, on the other hand, largely focus on the observed behaviors of targets in the real world. However, such methods may be lacking the ability to distill individual contextual factors that led to the value of correlated sub-indicators in the first place. We recommend that future studies identify linkages, causality, and contextual factors across all SDGs and at sub-indicator level, and not only at the target level. We also recommend that more attention be paid to linkage generation mechanisms and driven contextual factors, to avoid differences between methods. This should allow for a more comprehensive and deeper understanding of SDG linkages.

## Supplementary Information

Below is the link to the electronic supplementary material.

Supplementary file1 (XLSX 25 kb)

Supplementary file2 (DOCX 18 kb)

Supplementary file3 (XLSX 87 kb)

Supplementary file4 (XLSX 77 kb)

Supplementary file5 (DOCX 45 kb)
