# Supplementary material for: The role of context in identifying linkages between SDG 2 (food) and SDG 6 (water)
Source: Sustain Sci. 2022 May 30;17(4):1605–18. doi: 10.1007/s11625-022-01158-3 (PMC9338151; doi:10.1007/s11625-022-01158-3)
Supplement: Supplementary file 2 — Supplementary file2 (DOCX 18 kb) [file 11625_2022_1158_MOESM2_ESM.docx]

S2. Examples of text analysis of Voluntary National Reviews (VNRs)

To further explain the text analysis of VNRs, here are 4 text examples from the VNR of Afghanistan (GIRA, 2017).

Example 1

[In the beginning of SDG 2 section] *Life expectancy is low, infant under-five and maternal mortality are very high, and there is an extremely high prevalence of chronic malnutrition, poor sanitation, as well as a widespread occurrence of micronutrient deficiency diseases. The Nutrition Action Framework by the Ministry of Public Health (MoPH) seeks to* *address these issues in a coordinated, multi-sectoral fashion, However, despite ad-hoc arrangements/project based approaches, the initiative is dormant due to resource and capacity constraints.*

Key words are clearly and explicitly mentioned for SDG 2.2 (malnutrition) and SDG 6.2 (sanitation). They appear in one paragraph and the synergy could be inferred from the text (“address these issues in a coordinated, multi-sectoral fashion”) even though the initiative failed. The policy intention is not obvious, so the driver of the linkage is based on other qualitative studies. It is the linkage failed to be deal with. Finally, the synergy between SDG 6.2 (response) and SDG 2.2 (driver) is identified which is not successful to be deal with.

Example 2

[In the beginning of SDG 2 section] *For Afghanistan, agriculture*…*By acquiring certified seeds and saplings, the yield of farmers can be greatly enhanced.* *Improving irrigation conveyance systems including on farm water management, crop management and pest control can further contribute to greater efficiency and lower costs.*

It mentions water and agricultural, however, no key word on SDG is explicitly and clearly mentioned, especially for water related target. No linkage is found here.

Example 3

[Target 2.4 section] … *the government of Afghanistan does not use these watersheds properly and scientifically thereafter it could proceed many environmental problems such as soil erosion, landslides, floods and appalling, avalanches and not attracting and final resulting in financial losses. For overcome of the challenge set Watershed and restore vegetation and create a belt of greenery on one of the sound management Watershed, which this Ministry management expects to reach from 7235 hectares to 116,000 hectares in 2030.*

Since this text appears in the section of target 2.4 and explicitly and clearly mentions watershed management (SDG 6.5). The policy is intended for target 2.4 for future. Finally, the expected synergy between SDG 6.5 (response) and SDG 2.4 (driver) is identified.

Example 4

[Target 2.4 section] … *New methods of irrigation which is used for rain-fed land allows the use of solar pumps for irrigation in the form of drops, spray and other appropriate means, to save water and rain.*

This text appears in the section of target 2.4. Different from example 2, it explicitly and clearly mentions the key word “save water” (SDG 6.4). The policy is intended for target 2.4 for future. The expected synergy between SDG 6.4 (response) and SDG 2.4 (driver) is identified.

**Reference**

GIRA (2017) Voluntary National Review at the High Level Political Forum. SDGs’ Progress Report Afghanistan. Ministry of Economy, Government of the Islamic Republic of Afghanistan (GIRA)
