# Supplementary material for: The role of context in identifying linkages between SDG 2 (food) and SDG 6 (water)
Source: Sustain Sci. 2022 May 30;17(4):1605–18. doi: 10.1007/s11625-022-01158-3 (PMC9338151; doi:10.1007/s11625-022-01158-3)
Supplement: Supplementary file 5 — Supplementary file5 (DOCX 45 kb) [file 11625_2022_1158_MOESM5_ESM.docx]

**S5 The comparison with quantitative studies country by country**

Data for the correlation analysis is based on the UN SDG database (UNSD, 2020) downloaded on 17^th^ May 2020. The list of Voluntary National Reviews (VNRs) can be found in S1. The detailed text could be found in S3.

| **Linkage** | SDG 2.1 & SDG 6.2 | | |
| --- | --- | --- | --- |
| **Country** | Croatia | | |
| **Text** | Synergy | Correlation | Synergy |
| **Evidence**   1. The text is about the supply of food and hygiene for the most deprived people. 2. The text describes what is implemented. 3. The correlation analysis indicates:  \|  \| Undernourished people (% and millions) (SDG 2.1) \| \| --- \| --- \| \| People using safely managed sanitation, by urban/total (%) (SDG 6.2) \| Synergy \| \| People practicing open defecation by total (%) (SDG 6.2) \| Synergy \|  1. The data shows:  \|  \| Trend \| \| --- \| --- \| \| People using safely managed sanitation, by urban/total (%) (SDG 6.2) \| A marginal increasing trend \| \| People practicing open defecation, by total (%) (SDG 6.2) \| A marginal decreasing trend \| \| Undernourished people (% and millions) (SDG 2.1) \| A marginal decreasing trend \|  1. The prevalence of undernourishment is low (<2.5% since 2005); the proportion of population practicing open defecation is low (<0.6% in both rural and urban area); the proportion of the population using safely managed sanitation services is medium (around 60%). | | | |
| **Explanation**  Even though the synergy is statistically significant, the data also indicate the marginal improvements in the respective targets. The marginal improvements are partly attributed to the satisfying performance in some indicators. For the remaining indicators, like the proportion of the population using safely managed sanitation services, it is only improved by 0.26% from 2000 to 2017. However, the slow process does not contradict the text which describes the action for a part of people in the country. To summarize, evidence from text analysis and correlation analysis support each other. | | | |

| **Linkage** | SDG 2.2 & SDG 6.2 | | |
| --- | --- | --- | --- |
| **Country** | Guinea | | |
| **Text** | Synergy | Correlation | Non-classified |
| **Evidence**   1. The text describes the relationship between the high mortality of children under 5 and poor access to hygienic toilets. 2. The text describes what is expected or recognized; related intervention is not explicitly mentioned. 3. The correlation analysis indicates:  \|  \| Children moderately or severely wasted/stunted (% and thousands) (SDG 2.2) \| \| --- \| --- \| \| Population practicing open defecation, by urban/rural (%) (SDG 6.2) \| No statistically significant relationship \|  1. The data shows:  \|  \| Trend \| \| --- \| --- \| \| Population practicing open defecation, by urban/rural (%) (SDG 6.2) \| A steady decreasing trend \| \| Children moderately or severely wasted/stunted (% and thousands) (SDG 2.2) \| Fluctuations \|  1. According to the VNR of Guinea (GRG, 2018), factors that contribute to the fluctuation of SDG 2.2 include not only infectious disease and sanitation but also food supply, which is then affected by household financial situation, physical access to food, food stocks. Food sellers are the most vulnerable. | | | |
| **Explanation**  Text analysis and correlation analysis is different. Phase of intervention could explain the difference: the text describes what is expected or recognized and data shows what happened in the past. Moreover, SDG 2.2 is affected by other factors besides SDG 6.2.  The phase of intervention and food security situation (social-economic conditions) are the contextual factors that may affect linkages identification in this case. | | | |

| **Linkage** | SDG 2.2 & SDG 6.2 | | |
| --- | --- | --- | --- |
| **Country** | Indonesia | | |
| **Text** | Synergy | Correlation | Mix |
| **Evidence**   1. The text describes the relationship between the children's malnutrition and access to sanitation. 2. The text describes what is under development and not well integrated. 3. The correlation analysis indicates:  \|  \| Children moderately or severely wasted/stunted (% and thousands) (SDG 2.2) \| Children moderately or severely overweighted (% and thousands) (SDG 2.2) \| \| --- \| --- \| --- \| \| Population practicing open defecation, by urban/rural (%) (SDG 6.2) \| No statistically significant relationship \| Trade-offs \|  1. The data shows:  \|  \| Trend \| \| --- \| --- \| \| Population practicing open defecation, by urban/rural (%) (SDG 6.2) \| A decreasing trend with fluctuations \| \| Children moderately or severely wasted/stunted (% and thousands) (SDG 2.2) \| A steady increasing trend before 2010 and then a steady decreasing trend by 2018 \| \| Children moderately or severely overweighted (% and thousands) (SDG 2.2) \| Fluctuations \|  1. The problems of overweighted and wasted/stunted are affected by many different factors, and sanitation is one of them (WorldBank, 2015).   In general, stunting links more closely with sanitation than overweighting (WHO, 2020). | | | |
| **Explanation**  The text describes what is under development and data shows what happened in the past. The focused phase of intervention can explain part of the differences. Besides it, the population practicing open defecation (%) (SDG 6.2) seems not the determining factor of SDG 2.2. Other influencing factors for SDG2.2 include social norms, lifestyle, food availability.  The focused phase of intervention, social-economic conditions (social norms, lifestyle, food availability) are the contextual factor that affects the linkage identification in this case. | | | |

| **Linkage** | SDG 2.1 & SDG 6.1; SDG 2.1 & SDG 6.2 | | |
| --- | --- | --- | --- |
| **Country** | Kyrgyzstan | | |
| **Text** | Synergy | Correlation | Synergy |
| **Evidence**   1. The text describes the relationship between the nutrition supply and access to clean drinking water and sanitation. 2. The text describes what is expected or recognized. 3. The correlation analysis indicates:  \|  \| Undernourished people (% and millions) (SDG 2.1) \| \| --- \| --- \| \| Population using safely managed drinking water services/basic handwashing facilities on premises, by urban/rural (%) (SDG 6.1) \| Synergy \| \| Population practicing open defecation, by urban/rural (%) (SDG 6.2) \| Synergy \|  1. The data shows:  \|  \| Trend \| \| --- \| --- \| \| Population using safely managed drinking water services/basic handwashing facilities on premises, by urban/rural (%) (SDG 6.1) \| A steady increasing trend \| \| Population practicing open defecation, by urban/rural (%) (SDG 6.2) \| A steady decreasing trend and reaching zero after 2012 \| \| Undernourished people (% and millions) (SDG 2.1) \| A steady decreasing trend \| | | | |
| **Explanation**  Text analysis and correlation analysis indicate the same type of linkage. Since the text describes what is expected or recognized and data shows what happened in the past, evidence indicates both text analysis and correlation analysis hold by itself. | | | |

| **Linkage** | SDG 2.2 & SDG 6.2 | | |
| --- | --- | --- | --- |
| **Country** | Malawi | | |
| **Text** | Synergy | Correlation | Mix |
| **Evidence**   1. The text describes the relationship between malnutrition and access to sanitation for school children. 2. The text describes what is implemented. 3. The correlation analysis indicates:  \|  \| Proportion of children moderately or severely stunted/wasted/overweighted (%) (SDG 2.2) \| Children moderately or severely stunted/wasted/overweighted (thousands or millions) (SDG 2.2) \| \| --- \| --- \| --- \| \| Population practicing open defecation, by urban/rural (%) (SDG 6.2) \| Synergy \| No statistically significant relationship \| \| Proportion of population with basic handwashing facilities on premises, by urban/rural (%) (SDG 6.2) \| Trade-off \| No statistically significant relationship \|  1. The data shows:  \|  \| Trend \| \| --- \| --- \| \| Population practicing open defecation, by urban/rural (%) (SDG 6.2) \| A steady decreasing trend \| \| Proportion of population with basic handwashing facilities on premises, by urban/rural (%) (SDG 6.2) \| A steady decreasing trend \| \| Proportion of children moderately or severely stunted/wasted/overweighted (%) (SDG 2.2) \| A fluctuating decreasing trend and the absolute number varies larger than the percentage \| \| Children moderately or severely stunted/wasted/overweighted (thousands or millions) (SDG 2.2) \|  1. According to the VNR of Malawi (GRMw, 2020), efforts have been made to improve handwashing facilities. The lack of water and staff in the WASH sector weakens these efforts.   Factors that contribute to malnutrition (SDG 2.2) include food availability, diet, and infectious diseases (UNICEF, 2018). The last one is affected by sanitation (SDG 6.2). | | | |
| **Explanation**  Overall, evidence suggests a synergy between SDG 2.2 and SDG 6.2. The text from the VNR describes a project-level effect focusing on school children which may not have been scaled up to the country level. This may explain the decrease in access to handwashing facilities. The difference in spatial scale and a targeted group of people may explain why correlation analysis got no synergy. Different results of proportion and number of children related indicator suggest population growth affect linkage identification. Another reason that helps to explain the results of correlation analysis is social-economic situations (food security). Given the decrease in access to handwashing facilities, the change of SDG 2.2 may be explained by the change in the food supply, open defecation practices.  The project scale (spatial scale and target group of people) and social-economic conditions (food security) are the contextual factor that affects the linkage identification in this case. | | | |

| **Linkage** | SDG 2.2 & SDG 6.2 | | |
| --- | --- | --- | --- |
| **Country** | Mauritania | | |
| **Text** | Synergy | Correlation | Non-classified |
| **Evidence**   1. The text describes the relationship between malnutrition and access to sanitation. 2. The text describes what is expected or recognized. 3. The correlation analysis indicates:  \|  \| Children moderately or severely stunted/wasted/overweighted (% and thousands or millions) (SDG 2.2) \| \| --- \| --- \| \| Population practicing open defecation, by urban/rural (%) (SDG 6.2) \| No statistically significant \| \| Proportion of population with basic handwashing facilities on premises, by urban/rural (%) (SDG 6.2) \| No statistically significant \|  1. The data shows:  \|  \| Trend \| \| --- \| --- \| \| Population practicing open defecation, by urban/rural (%) (SDG 6.2) \| A steady decreasing trend \| \| Proportion of population with basic handwashing facilities on premises, by urban/rural (%) (SDG 6.2) \| Stability \| \| Children moderately or severely stunted/wasted/overweighted (% and thousands or millions) (SDG 2.2) \| A decreasing trend with fluctuations \|  1. According to the VNR of Mauritania (GIRM, 2019), malnutrition is affected by food security, access to drinking water and sanitation; the agricultural production is highly rainfall dependent; the acute malnutrition shows a significant seasonal pattern. | | | |
| **Explanation**  Overall, evidence suggests a synergy between SDG 2.2 and SDG 6.2. The change of SDG 2.2 may be explained by the change in food security, especially for acute malnutrition.  The social-economic conditions (food security, acute malnutrition because of unstable food production) are the contextual factor that affects the linkage identification in this case. | | | |

| **Linkage** | SDG 2.2 & SDG 6.2 | | |
| --- | --- | --- | --- |
| **Country** | Mozambique | | |
| **Text** | Synergy | Correlation | Mix |
| **Evidence**   1. The text describes the relationship between malnutrition and access to sanitation and hygiene. 2. The text describes what is implemented. 3. The correlation analysis indicates:  \|  \| Proportion of children moderately or severely stunted (%) (SDG 2.2) \| Children moderately or severely stunted (thousands) (SDG 2.2) \| Children moderately or severely wasted/overweighted (thousands and %) (SDG 2.2) \| \| --- \| --- \| --- \| --- \| \| Proportion of population practicing open defecation/using safely managed sanitation services, by urban/rural (%) (SDG 6.2) \| Synergy \| Trade-off \| No statistically significant relationship \|  1. The data shows:  \|  \| Trend \| \| --- \| --- \| \| Proportion of population practicing open defecation, by urban/rural (%) (SDG 6.2) \| A steady decreasing trend \| \| Proportion of population using safely managed sanitation services, by urban/rural (%) (SDG 6.2) \| A steady increasing trend \| \| Proportion of children moderately or severely stunted (%) (SDG 2.2) \| A steady decreasing trend \| \| Children moderately or severely stunted (thousands) (SDG 2.2) \| A steady increasing trend \| \| Children moderately or severely wasted/overweighted (thousands and %) (SDG 2.2) \| Fluctuations \|  1. According to the VNR of Mozambique (GRMz, 2020), other factors, like food insecurity, conservative cultural habits, diet, food production behavior contribute to malnutrition.   In general, stunting links more closely with sanitation than wasting and overweighting because stunting reflects chronic malnutrition (WHO, 2020). | | | |
| **Explanation**  Synergies and trade-offs are suggested by correlation analysis at the same time. Population growth is the only different factor in terms of the number and proportion of children moderately or severely stunted. Synergies are suggested by the text and supported by correlation analysis. From correlation analysis, synergies exist between proportion of population practicing open defecation/using safely managed sanitation services (%) (SDG 6.2) and proportion of children moderately or severely stunted (%) (SDG 2.2). Indicator for stunted children reflects chronic malnutrition which is affected by the spread of infectious disease and long-term food security. Thus, these synergies support the results of text analysis. The change in children moderately or severely wasted/overweighted (thousands and %) maybe because of other social-economic factors.  The social-economic conditions (population growth, food security, conservative cultural habits, diet, food production behavior) are the contextual factor that affects the linkage identification in this case. | | | |

| **Linkage** | SDG 2.1 & SDG 6.1; SDG 2.2 & SDG 6.1; SDG 2.2 & SDG 6.2 | | |
| --- | --- | --- | --- |
| **Country** | Nepal | | |
| **Text** | Synergy | Correlation | Mix |
| **Evidence**   1. The text describes the relationship between hunger, child malnutrition and access to water, sanitation, and hygiene. 2. The text describes what is expected or recognized. 3. The correlation analysis indicates:  \|  \| Undernourished people (millions and %) (SDG 2.1) \| Proportion of children moderately or severely wasted/overweight (%) (SDG 2.2) \| Children moderately or severely stunted/wasted (thousands) (SDG 2.2) \| Children moderately or severely overweight (thousands) (SDG 2.2) \| Proportion of children moderately or severely stunted (%) (SDG 2.2) \| \| --- \| --- \| --- \| --- \| --- \| --- \| \| Proportion of population using safely managed drinking water services (rural/all, %) (SDG 6.1) \| Synergy \| No statistically significant relationship \| Synergy \| No statistically significant relationship \| Synergy \| \| proportion of population using safely managed drinking water services (urban, %) (SDG 6.1) \| Trade-off \| No statistically significant relationship \| Trade-off \| No statistically significant relationship \| Trade-off \| \| Proportion of population practicing open defecation, by urban/rural (%) (SDG 6.2) \|  \| No statistically significant relationship \| Synergy \| No statistically significant relationship \| Synergy \|  1. The data shows:  \|  \| Trend \| \| --- \| --- \| \| Proportion of population using safely managed drinking water services (rural/all, %) (SDG 6.1) \| A steady increasing trend \| \| proportion of population using safely managed drinking water services (urban, %) (SDG 6.1) \| A steady decreasing trend \| \| Proportion of population practicing open defecation, by urban/rural (%) (SDG 6.2) \| A steady decreasing trend \| \| Undernourished people (millions and %) (SDG 2.1) \| A steady decreasing trend \| \| Proportion of children moderately or severely wasted/overweight (%) (SDG 2.2) \| Fluctuations \| \| Children moderately or severely stunted/wasted (thousands) (SDG 2.2) \| A steady decreasing trend \| \| Children moderately or severely overweight (thousands) (SDG 2.2) \| An increasing trend with fluctuations \| \| Proportion of children moderately or severely stunted (%) (SDG 2.2) \| A steady decreasing trend \|  1. Nepal is undergoing double malnutrition where both overweight and underweight exist (Schwinger et al., 2020). Underweight children are generally in rural areas (GN, 2020) and overweight is much more popular in an urban area (Karki et al., 2019). Unbalanced diet and lack of physical exercise contribute to the overweight problem (Karki et al., 2019).   A sizeable people in the urban area rely on untreated water from local springs, streams, and wells whose quantity and quality are threatened by climate change, pollution, population growth (Pandey, 2020).  Food insecurity contributes to the wasting problem (Nepali et al., 2020).  In general, stunting links more closely with sanitation than wasting and overweighting because stunting reflects the chronic malnutrition (WHO, 2020). | | | |
| **Explanation**  First, in general, the text describes what is expected or recognized and data shows what happened in the past.  For the linkage between SDG 6.1 and SDG 2.1, the text analysis and correlation analysis support each other except for the case of drinking water in an urban area. Reasons for this difference include that the undernourishment seems prevalent in the rural area even though no data explicitly show the proportion of undernourishment in the rural and urban area separately.  The difference between SDG 2.2 (in terms of stunted children) and SDG 6.1 (in terms of drinking water in the urban and rural area separately) can be explained by the same reason above, i.e. stunted children are mainly found in a rural area and conducting correlation with drinking water in an urban area does not make sense.  Synergies between SDG 6.2 (in terms of the population practicing open defecation) and SDG 2.2 (in terms of stunted children) support the text. Population growth, social-economic situations (like food security, unbalanced diet, and lack of physical exercise) are the external factors that affect the change of SDG 2.2 in terms of wasted/overweighted.  In this case, focused phase of intervention, project scale (targeted group of people), and social-economic conditions (like population growth, food security, unbalanced diet, and lack of physical exercise) are the contextual factors that affect the linkage identification. | | | |

| **Linkage** | SDG 2.2 & SDG 6.1 | | |
| --- | --- | --- | --- |
| **Country** | Peru | | |
| **Text** | Synergy | Correlation | Synergy |
| **Evidence**   1. The text describes the relationship between chronic child malnutrition and access to clean drinking water. 2. The text describes what is implemented. 3. The correlation analysis indicates:  \|  \| Children moderately or severely wasted/stunted/overweighted (thousands and %) (SDG 2.2) \| \| --- \| --- \| \| Proportion of population using safely managed drinking water services, by urban/rural (%) (SDG 6.1) \| Synergy \|  1. The data shows:  \|  \| Trend \| \| --- \| --- \| \| Proportion of population using safely managed drinking water services, by urban/rural (%) (SDG 6.1) \| A steady increasing trend \| \| Children moderately or severely wasted/stunted/overweighted (thousands and %) (SDG 2.2) \| A steady decreasing trend with a little fluctuation \| | | | |
| **Explanation**  Results from text analysis and correlation analysis support each other. | | | |

| **Linkage** | SDG 2.2 & SDG 6.2 | | |
| --- | --- | --- | --- |
| **Country** | Timor-Leste | | |
| **Text** | Synergy | Correlation | Non-classified |
| **Evidence**   1. The text describes the relationship between child malnutrition, especially stunting, and access the sanitation and hygiene, e.g. defecation practices and handwashing. 2. The text describes what is implemented, observed, and expected. 3. The correlation analysis indicates:  \|  \| Children moderately or severely stunted/wasted (thousands and %) (SDG 2.2) \| \| --- \| --- \| \| Proportion of population practicing open defecation, by urban/rural (%) (SDG 6.2) \| No statistically significant relationship \|  1. The data shows:  \|  \| Trend \| \| --- \| --- \| \| Proportion of population practicing open defecation, by urban/rural (%) (SDG 6.2) \| A steady decreasing trend \| \| Children moderately or severely stunted/wasted (thousands and %) (SDG 2.2) \| Fluctuations \|  1. While access to water and sanitation is an influencing factor for the achievement of SDG 2.2, according to the VNR of Timor-Leste (GTL, 2019), other factors are also identified. For example, diets of women of reproductive age, teenage pregnancy, sexual and reproductive health, food security, gender equity. All of them play an important role in some cases. | | | |
| **Explanation**  The synergy between malnutrition and sanitation is well recognized and incorporated into national action. The correlation analysis reports no synergy. The difference can be explained by the exclusion of other factors that affect SDG 2.2.  Social-economic conditions (e.g. diets of women of reproductive age, teenage pregnancy, sexual and reproductive health, food security, gender equity) are the contextual factor that affects the linkage identification in this case. | | | |

| **Linkage** | SDG 2.1 & SDG 6.1 | | |
| --- | --- | --- | --- |
| **Country** | Uganda | | |
| **Text** | Synergy | Correlation | Mix |
| **Evidence**   1. The text describes the relationship between food security and drinking water security, targeted mainly at female refugees on a small scale. 2. The text describes what is implemented during 2017 and 2019. 3. The correlation analysis indicates:  \|  \| Prevalence of undernourishment (millions, %) (SDG 2.1) \| \| --- \| --- \| \| Proportion of population using safely managed drinking water services (urban, %) (SDG 6.1) \| Synergy \| \| Proportion of population using safely managed drinking water services, by rural/total (%) (SDG 6.1) \| Trade-off \|  1. The data shows:  \|  \| Trend \| \| --- \| --- \| \| Proportion of population using safely managed drinking water services (urban, %) (SDG 6.1) \| A steady decreasing trend \| \| Proportion of population using safely managed drinking water services, by rural/total (%) (SDG 6.1) \| A steady increasing trend \| \| Prevalence of undernourishment (millions, %) (SDG 2.1) \| A steady increasing trend \|  1. According to the VNR of Slovenia (GRS, 2020), Uganda has received many refugees who suffer from sever food insecurity and resident near the capital. | | | |
| **Explanation**  First, refugee is the promising reason to explain the contradicted trend of the coverage of drinking water in the urban and rural area. Given that refugee also threats the achievement of SDG 2.1 and resident near the city, the correlation analysis makes sense. Even though the correlation analysis indicates synergy, the trends are unexpected and both SDG 6.2 and SDG 2.1 (urban) are mainly driven by the social-economic conditions (refugee). Text analysis indicates a synergy; however, the scale of the project is small, and it may not have been scaled up to the country level.  The project scale and social-economic conditions (refugee) are the contextual factors that affect linkages identification. | | | |

**Reference**

GIRM (2019) Revue Nationale Volontaire République Islamique de Mauritanie. Stratégie de Croissance Accélérée et de Prospérité Partagée, Government of the Islamic Republic of Mauritania (GIRM)

GN (2020) Nepal National review of Sustainable Development Goals. National Planning Commission, Government of Nepal (GN), Kathmandu

GRG (2018) Contribution nationale volontaire à la mise en œuvre des odd au forum politique de haut niveau. Government of the Republic of Guinea (GRG)

GRMw (2020) Malawi 2020 Voluntary National Review report for Sustainable Development Goals (SDGs). Government of the Republic of Malawi (GRMw)

GRMz (2020) Report Voluntary National Review of Agenda 2030 for Sustainble Development. Government of the Republic of Mozambique (GRMz)

GRS (2020) Implementation of the Sustainable Development Goals. Second Voluntary National Review Slovenia 2020. Government of the Republic of Slovenia (GRS)

GTL (2019) Report on the Implementation of the Sustainable Development Goals: From ashes to reconciliation, reconstruction and sustainable development, Voluntary National Review of Timor-Leste 2019. Government of Timor-Leste (GTL), Dili, Timor-Leste

Karki A, Shrestha A, Subedi N (2019) Prevalence and associated factors of childhood overweight/obesity among primary school children in urban Nepal. BMC Public Health 19:1055. doi:10.1186/s12889-019-7406-9

Nepali S, Simkhada P, Davies IG (2020) Association between wasting and food insecurity among children under five years: findings from Nepal demographic health survey 2016. BMC Public Health 20:1027. doi:10.1186/s12889-020-09146-x

Pandey CL (2020) Managing urban water security: challenges and prospects in Nepal. Environment, Development and Sustainability. doi:10.1007/s10668-019-00577-0

Schwinger C, Chandyo RK, Ulak M, Hysing M, Shrestha M, Ranjitkar S, Strand TA (2020) Prevalence of Underweight, Overweight, and Obesity in Adults in Bhaktapur, Nepal in 2015-2017. Front Nutr 7:567164. doi:10.3389/fnut.2020.567164

UNICEF (2018) Nutrition statistics in Malawi. <https://www.unicef.org/malawi/sites/unicef.org.malawi/files/2018-09/UNICEF_Nutrition_Factsheet_2018.pdf> Accessed 2-dec 2020

UNSD (2020) United Nations Global SDG Database. <https://unstats.un.org/sdgs/indicators/database/>

WHO (2020) Malnutrition. <https://www.who.int/news-room/fact-sheets/detail/malnutrition> Accessed 5-Dec-2020

WorldBank (2015) The Double Burden of Malnutrition in Indonesia. <https://www.worldbank.org/en/news/feature/2015/04/23/the-double-burden-of-malnutrition-in-indonesia#:~:text=In%20Indonesia%2C%208.4%20million%20children,the%20double%20burden%20of%20malnutrition>. Accessed 5-Dec-2020
